# Warm ischemia time, postreperfusion syndrome and initial poor function after liver transplantation: are they connected?

**DOI:** 10.1186/cc14458

**Published:** 2015-03-16

**Authors:** E Scarlatescu, G Manga, G Droc, D Tomescu

**Affiliations:** 1 Fundeni Clinical Institute, Bucharest, Romania

## Introduction

Factors associated with initial poor graft function (IPGF) after liver transplantation are still under debate. Although the initial insult to the graft begins during the cold ischemia time (CIT), recent studies showed that most injuries occur during rewarming. Ischemic- reperfusion (I/R) injuries are present in all grafts and may be responsible for postoperative graft dysfunction. Along with other factors, I/R injury may also play a role in the development of postreperfusion syndrome (PRS) after revascularization of the liver graft. The aim of this study was to assess whether longer warm ischemia time (WIT) is associated with PRS or with IPGF after liver transplant. The secondary aim was to investigate whether patients with intraoperative PRS have a higher risk for postoperative IPGF.

## Methods

This retrospective observational study included 60 liver transplant patients. We excluded from the study group patients with retransplant procedures, and the recipients of divided grafts and of grafts from extended criteria donors. We recorded: demographic data, intraoperative PRS, CIT, WIT, ALT, AST levels and standard coagulation tests on postoperative days (POD) 1 to 5. Statistical analysis was performed using SPSS Statistics v.19.1 with significant *P *value under 0.05.

## Results

We used the criteria of Nanashima and colleagues for the diagnosis of IPGF (ALT and/or AST level above 1,500 IU/l within 72 hours after OLT). The study group included 33 men (55%) and 27 women. Mean (±SD) age was 50.56 (±13.26) years. WIT longer than 60 minutes correlated significantly with ALT and AST levels in POD 1 to 3 (*P *< 0.0001 for ALT in POD 1 to 3, *P *= 0.001 for AST in POD 1, *P *= 0.007 and 0.013 for AST in POD 2 and 3) and with prothrombin time (*P *= 0.008 in POD 1, *P *= 0.03 in POD 2 and *P *= 0.015 in POD 3). We could not find a correlation between PRS and WIT (*P *= 0.566), CIT (*P *= 0.439) or transaminase levels on POD 1 to 3. The correlation between WIT >60 minutes and IPGF was confirmed using the Pearson chi-square test (*P *< 0.0001). The same test was used to correlate IPGF with PRS with nonsignificant results (*P *= 0.876).

## Conclusion

Our study showed that PRS is not a risk factor for IPGF after liver transplantation. WIT over 60 minutes does not influence the development of PRS, but is associated with IPGF after liver transplantation. Close monitoring of liver tests in the early postoperative period is very important especially in recipients of grafts with WIT over 60 minutes. Further efforts to decrease WIT may prove useful for the reduction of IPGF in liver transplant patients.

